# Spatial-temporal analysis of hepatitis E in Hainan Province, China (2013-2022): insights from four major hospitals

**DOI:** 10.3389/fpubh.2024.1381204

**Published:** 2024-06-27

**Authors:** Zhi Yun, Panpan Li, Jinzhong Wang, Feng Lin, Wenting Li, Minhua Weng, Yanru Zhang, Huazhi Wu, Hui Li, Xiaofang Cai, Xiaobo Li, Xianxian Fu, Tao Wu, Yi Gao

**Affiliations:** ^1^Department of Infectious Diseases, Affiliated Hainan Hospital of Hainan Medical University (Hainan General Hospital), Haikou, China; ^2^Intensive Care Unit, The Second Affiliated Hospital of Hainan Medical University, Haikou, China; ^3^Department of Infectious Diseases, The Second Affiliated Hospital of Hainan Medical University, Haikou, China; ^4^Department of Neurosurgery, Haikou Municipal People's Hospital and Central South University Xiangya Medical College Affiliated Hospital, Haikou, China; ^5^Clinical Lab, Haikou Municipal People’s Hospital and Central South University Xiangya Medical College Affiliated Hospital, Haikou, China; ^6^National Health Commission Key Laboratory of Tropical Disease Control, Hainan Medical University, Haikou, China

**Keywords:** hepatitis E, trend analysis, spatial autocorrelation, forecasting model, Hainan Province

## Abstract

**Objective:**

Exploring the Incidence, Epidemic Trends, and Spatial Distribution Characteristics of Sporadic Hepatitis E in Hainan Province from 2013 to 2022 through four major tertiary hospitals in the Province.

**Methods:**

We collected data on confirmed cases of hepatitis E in Hainan residents admitted to the four major tertiary hospitals in Haikou City from January 2013 to December 2022. We used SPSS software to analyze the correlation between incidence rate and economy, population density and geographical location, and origin software to draw a scatter chart and SAS 9.4 software to conduct a descriptive analysis of the time trend. The distribution was analyzed using ArcMap 10.8 software (spatial autocorrelation analysis, hotspot identification, concentration, and dispersion trend analysis). SAS software was used to build an autoregressive integrated moving average model (ARIMA) to predict the monthly number of cases in 2023 and 2024.

**Results:**

From 2013 to 2022, 1,922 patients with sporadic hepatitis E were treated in the four hospitals of Hainan Province. The highest proportion of patients (*n* = 555, 28.88%) were aged 50–59 years. The annual incidence of hepatitis E increased from 2013 to 2019, with a slight decrease in 2020 and 2021 and an increase in 2022. The highest number of cases was reported in Haikou, followed by Dongfang and Danzhou. We found that there was a correlation between the economy, population density, latitude, and the number of cases, with the correlation coefficient |r| value fluctuating between 0.403 and 0.421, indicating a linear correlation. At the same time, a scatter plot shows the correlation between population density and incidence from 2013 to 2022, with r^2^ values fluctuating between 0.5405 and 0.7116, indicating a linear correlation. Global Moran’s I, calculated through spatial autocorrelation analysis, showed that each year from 2013 to 2022 all had a Moran’s I value >0, indicating positive spatial autocorrelation (*p* < 0.01). Local Moran’s I analysis revealed that from 2013 to 2022, local hotspots were mainly concentrated in the northern part of Hainan Province, with Haikou, Wenchang, Ding’an, and Chengmai being frequent hotspot regions, whereas Baoting, Qiongzhong, and Ledong were frequent cold-spot regions. Concentration and dispersion analysis indicated a clear directional pattern in the average density distribution, moving from northeast to southwest. Time-series forecast modeling showed that the forecast number of newly reported cases per month remained relatively stable in 2023 and 2024, fluctuating between 17 and 19.

**Conclusion:**

The overall incidence of hepatitis E in Hainan Province remains relatively stable. The incidence of hepatitis E in Hainan Province increased from 2013 to 2019, with a higher clustering of cases in the northeast region and a gradual spread toward the southwest over time. The ARIMA model predicted a relatively stable number of new cases each month in 2023 and 2024.

## Introduction

Hepatitis E virus (HEV) is a spherical viral particle with a diameter of approximately 27–34 nm. It has spikes and indentations on its surface (1). In 1983, it was discovered in a patient’s feces using immunoelectron microscopy (2). Acute HEV infection generally has a good prognosis, however, there is still the possibility of extrahepatic complications, such as neurological symptoms and kidney damage (3). During pregnancy, HEV infection increases the risk of adverse outcomes, particularly during the second and third trimesters. After infection with HEV genotypes 1 and 2, a significant proportion of pregnant women progress to acute liver failure, with a mortality rate of up to 15–25% (4). A prospective study found that pregnant women infected with HEV have worse outcomes than those with acute viral hepatitis caused by other hepatitis viruses (5). In immunocompromised individuals and those with concomitant chronic hepatitis B infection, HEV infection often imposes a greater burden with a significantly longer course, higher economic burden, and greater resultant health loss (6). The prevention and control of hepatitis E (HE) is a major public health issue (7–10).

According to the World Health Organization, an estimated 20 million new HEV infections occur worldwide each year, with over 3 million people experiencing symptoms and 56,600 deaths related to HEV infection. The prevalence of HEV infection is high in developing countries in Asia and Africa (11, 12). In these countries, HE accounts for more than half of all cases of acute hepatitis. In North America, Europe, and other countries, reported cases and localized HE outbreaks are increasing yearly (13). The seroprevalence of HEV antibodies in the European population ranges from 7.5 to 31.9% (14). HE is endemic in China. The incidence rate of HE in China has been gradually increasing and HE is the most frequent cause of acute viral hepatitis in China (15). According to the latest data from the Chinese Center for Disease and Prevention,^1^ from 2018 to 2021 the number of cases and deaths from HE were higher than those from hepatitis A. Therefore, it is important to explore the spatiotemporal distribution characteristics of HE. Spatial epidemiology has been widely applied in infectious disease research (16–18). Recently, it has been applied to predict various infectious diseases such as hand-foot-and-mouth disease (19), tuberculosis, and COVID-19 (20, 21). By integrating geographical location and time information, these models can reveal the patterns and influencing factors of disease transmission and can thus be used to prevent and control various legally reportable infectious diseases (22). The autoregressive integrated moving average (ARIMA) model is a statistical model used for time-series analysis and forecasting. This model has been widely applied to the study of Hantavirus hemorrhagic fever (23) and COVID-19 The study aimed to use the model to analyze the epidemiological data of patients with HE to understand the spatiotemporal dynamics and epidemiological characteristics of HE incidence in Hainan Province in China. In addition, this study aimed to forecast the monthly incidence in 2023 and 2024 using this model.

## Materials and methods

### Study subject source

**Figure 1:**
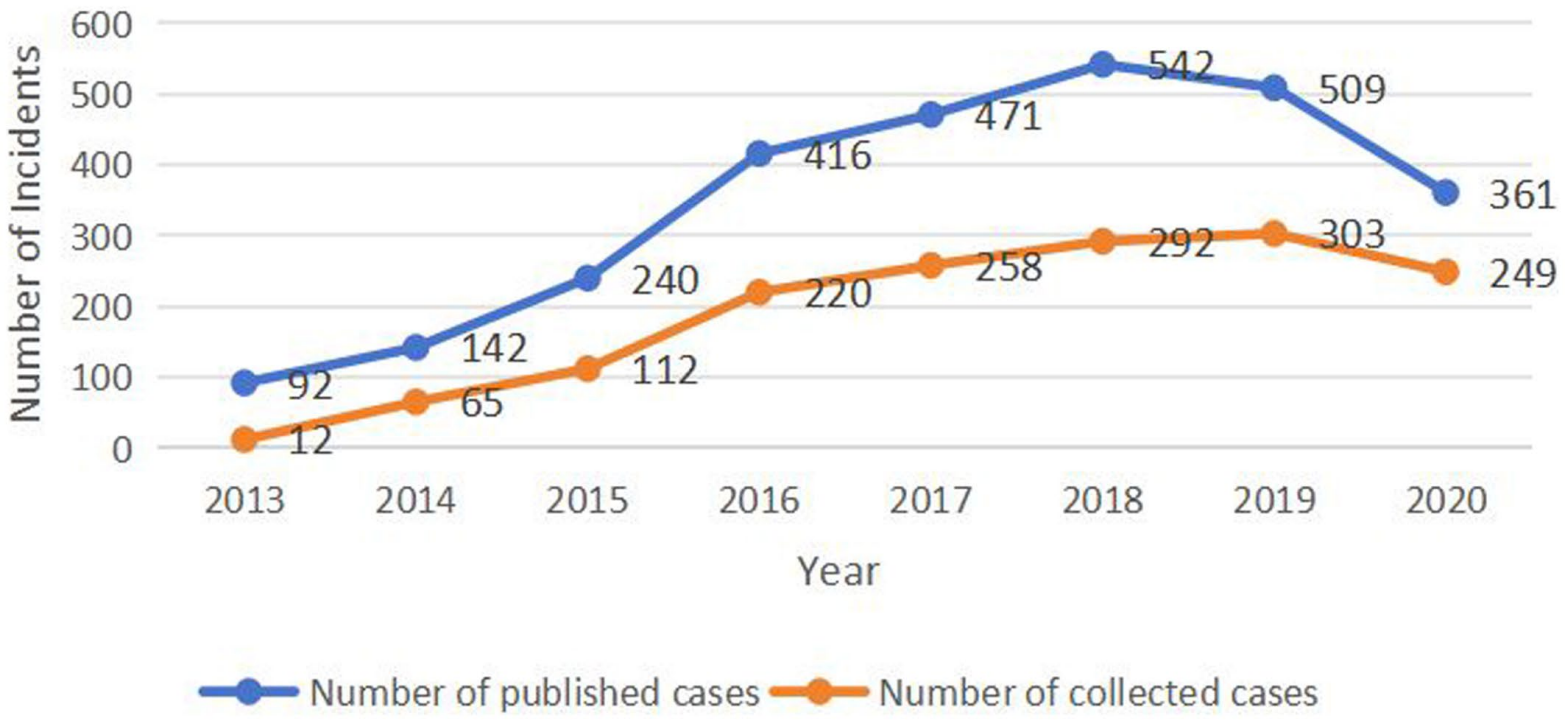
Comparison of the number of published and collected cases.

Data on confirmed cases of HE from January 2013 to December 2022 were collected from four tertiary hospitals in Haikou, Hainan Province. The data collected included age, sex, date of onset, date of admission, date of diagnosis, residential address, and liver function indicators. These hospitals in Hainan Province can detect HE and are also the four hospitals with the highest number of admitted cases in the past decade. According to the comparison of the incidence data collected and the public health data (published up to 2020) released by the Chinese Center for Disease Control and Prevention,^2^ the data trends of both are the same. Among them, from 2016 to 2020, the number of cases in these four hospitals exceeded 50% of the cases in Hainan Province (Figure 1).

Demographic data were obtained from the Hainan Provincial Government Data Unified Open Platform.^3^ The electronic map source for the fourth-level coordinate boundary data for cities and counties in the province was obtained from the Hainan Provincial Geographic Information Public Service Platform.^4^ Population density and economic data from the 2023 Hainan Statistical Yearbook. The longitude and latitude data of each city and county are sourced from Tianmap.^5^ In this study, all cases of HE were categorized according to the 2018 ESAL Clinical Practice Guidelines for HE Infection (24), based on accurate diagnostic criteria.

### Compliance with ethics guidelines

The study was approved by the institutional review boards of each of the project implementation hospitals, and all procedures complied with the local ethical standards, and the Declaration of Helsinki of 1964 and subsequent amendments. As this was a retrospective observational study, the requirement for informed consent was waived.

### Data processing and analysis

The administrative code was used as the linking field to link the incidence data of each city and county in Hainan Province with its vector electronic map (*shop format) using ArcGIS software. IBM SPSS Statistics 24.0 was used to analyze the correlation between population density, economy, geographical location (longitude and latitude), and incidence rate, and origin software was used to draw scatter plots. Data management and statistical analysis, including logistic regression, were performed using SAS 9.4 (version 9.4, SAS Institute, Inc., Cary, NC, United States). Plots were constructed using R version 4.3.2 (R Foundation for Statistical Computing, Vienna, Austria). Categorical variables were constructed using frequencies and percentages. Between-group comparisons were performed using the chi-square test. Fisher’s exact test was used when the expected frequency in any cell was <5.

### Spatial–temporal aggregation and distribution characteristics of he from 2013 to 2022 and modeling prediction

ArcMap 10.8 was used for map drawing, spatial hotspot analysis, and spatial concentration and dispersion trend analysis of the HE epidemic. SAS 9.4 software was used to build an ARIMA model and the “auto. Arima” function in R was used to automatically search for the best parameters based on the Akaike information criterion (AIC), to determine the optimal model. The model was tested using the Ljung-Box test to assess the goodness of fit. Finally, a well-fitted model was used to predict the monthly incidence time series of HE in Hainan Province for 2023 and 2024. The specific indicators are described in the sections that follow.

### Global spatial autocorrelation analysis

The spatial patterns of the entire study area were explored by using a single value to reflect the autocorrelation pattern of the region. The primary indicator in the global autocorrelation analysis is Moran’s I index, with a range of values between −1 and 1. A value closer to 1 indicates a higher degree of clustering, whereas a value closer to −1 indicates greater dispersion. The formula is as follows:


$$I=\frac{n}{S_{0}}\times \frac{\sum_{i=1}^{n}\sum_{j=1}^{n}w_{ij}\left(y_{i}-\overset{↼}{y}\right)\left(y_{j}-\overset{↼}{y}\right)}{\sum_{i=1}^{n}{\left(y_{i}-\overset{↼}{y}\right)}^{2}}$$

In this context, $S_{0}$ = $\overset{n}{\underset{i=1}{\sum }}\overset{n}{\underset{j=1}{\sum }}w_{ij}$ , where n represents the total number of cities and counties, $y_{i}$ and $y_{j}$ represent the incidence numbers of the ith and jth spatial units, respectively, and $\overset{↼}{y}$ represents the mean incidence of cases across all cities and counties and serves as the spatial weighting value. The global Moran’s I require a significance test, typically a Z-test. The formula for the Z-test of the global Moran’s I is as follows:


$$Z=\frac{I-E\left(I\right)}{\sqrt{\mathrm{var}\left(I\right)}}$$

Where the calculation methods for the mean and variance are:


$$E\left(I\right)=-\frac{1}{n-1}$$

$$Var\left(I\right)=\frac{\begin{array}{l} n^{2}\left(n-1\right)\frac{1}{2}\sum_{i\neq j}{\left(w_{ij}+w_{ji}\right)}^{2}-n\left(n-1\right)\sum_{k}\\ {\left(\sum_{j}w_{kj}+\sum_{i}w_{ik}\right)}^{2}-2{\left(\sum_{i\neq j}w_{ij}\right)}^{2} \end{array}}{\left(n+1\right){\left(n-1\right)}^{2}{\left(\sum_{i\neq j}w_{ij}\right)}^{2}}$$

#### Local Moran’s I analysis

Local Moran’s I analysis was used to study the degree of correlation between the attributes of each spatial unit and its neighboring units. It can effectively detect spatial variation caused by spatial correlation, complementing the limitations of the global Moran’s I analysis and identifying spatial hot spots or high-incidence areas. The specific indicator was the local Moran’s I index. For each calculated value, its standardized Z-value was computed for significance testing, determining whether it passed the test and identifying hot spots and cold spots. In this study, Z-value >2.58 indicated hotspots, and Z-values <2.58 indicated cold-spots. *p*-values <0.1, < 0.05, and < 0.01 corresponded to the 90, 95, and 99% confidence levels, respectively. The results were visually presented using ArcMap software in the form of a local indicator of a spatial association (LISA) cluster map. The formula is as follows:


$$I_{i}=\frac{Z_{i}}{S^{2}}\overset{n}{\underset{j\neq i}{\sum }}w_{ij}Z_{j}$$

In this context, $Z_{i}=y_{i}-\overset{↼}{y},Z_{j}=y_{j}-\overset{↼}{y},S^{2}=\frac{1}{n}\sum {\left(y_{i}-\overset{↼}{y}\right)}^{2}$ , where $w_{ij}$ represents spatial weighting values, n represents the total number of all cities and counties, $I_{i}$ represents the local Moran’s I index for the i-th city or county, $y_{i}$ represents the incidence of HE in the i-th city or county, $y_{j}$ represents the incidence of HE in the j-th city or county, and $\overset{↼}{y}$ represents the average incidence of HE across all cities and counties. The local Moran’s I require a Z-test, and the formula for the Z-test is:


$$Z\left(I_{i}\right)=\frac{I_{i}-E\left(I_{i}\right)}{\sqrt{Var\left(I_{i}\right)}}$$

In this context, $\left(I_{i}\right)$ is the expected value of the local Moran’s I, and $Var\left(I_{i}\right)$ is the variance of the local Moran’s I. Based on the Z-value, the corresponding *p*-value is calculated to assess the local pattern for each city or county.

### Time-series analysis and forecasting

Time series can be represented as a set of data points arranged chronologically. The ARIMA model was used to predict the univariate time-series data. This model uses a combination of differencing, autoregression, and moving averages to estimate the peak time and magnitude of HE cases monthly and annually for each city and county in the province in 2023 and 2024. This included:

1. Data preparation: Checks were performed to determine whether the sequence satisfied stationarity and the augmented Dickey-Fuller test results for time-series stationarity were examined. The t-value was analyzed to determine whether the assumption of non-stationarity could be significantly rejected (*p* < 0.05). The data were inspected before and after differencing (d-value) to confirm stationarity (small fluctuation amplitudes).
2. Model order determination: The ARIMA model is represented as ARIMA (p, d, q), where p (autoregressive order), d (differencing order), and q (moving average order) are the three essential parameters. The d-value was determined by differentiating the time-series data in a certain order. The *p*-value was determined by examining the autocorrelation coefficient plots (ACF) for tailing and partial autocorrelation coefficient plots (PACF) for truncation. The q value was determined from the ACF truncation and PACF tailing.
3. Model parameter estimation: The AIC values were combined and the AIC values for different differencing orders were compared. The “auto. Arima” function in R was used to search for the best parameters based on the AIC (minimizing the value).
4. Model evaluation: Model residuals were visualized to check whether they follow a normal distribution (normality test). The *p*-value of the Q statistic (*p* > 0.01 indicates white noise) was used to test the model’s white noise; that is, the Ljung-Box test was used to assess whether the residual sequence was random and evaluate the model fitting effect.
5. Model prediction: The well-fitted ARIMA (0,1,1)(1,0,0)[12] model was used to forecast the monthly incidence time series of HE in Hainan Province for 2023 and 2024. The predicted results were plotted to visualize the forecast.

## Results

### He incidence

**Figure 2:**
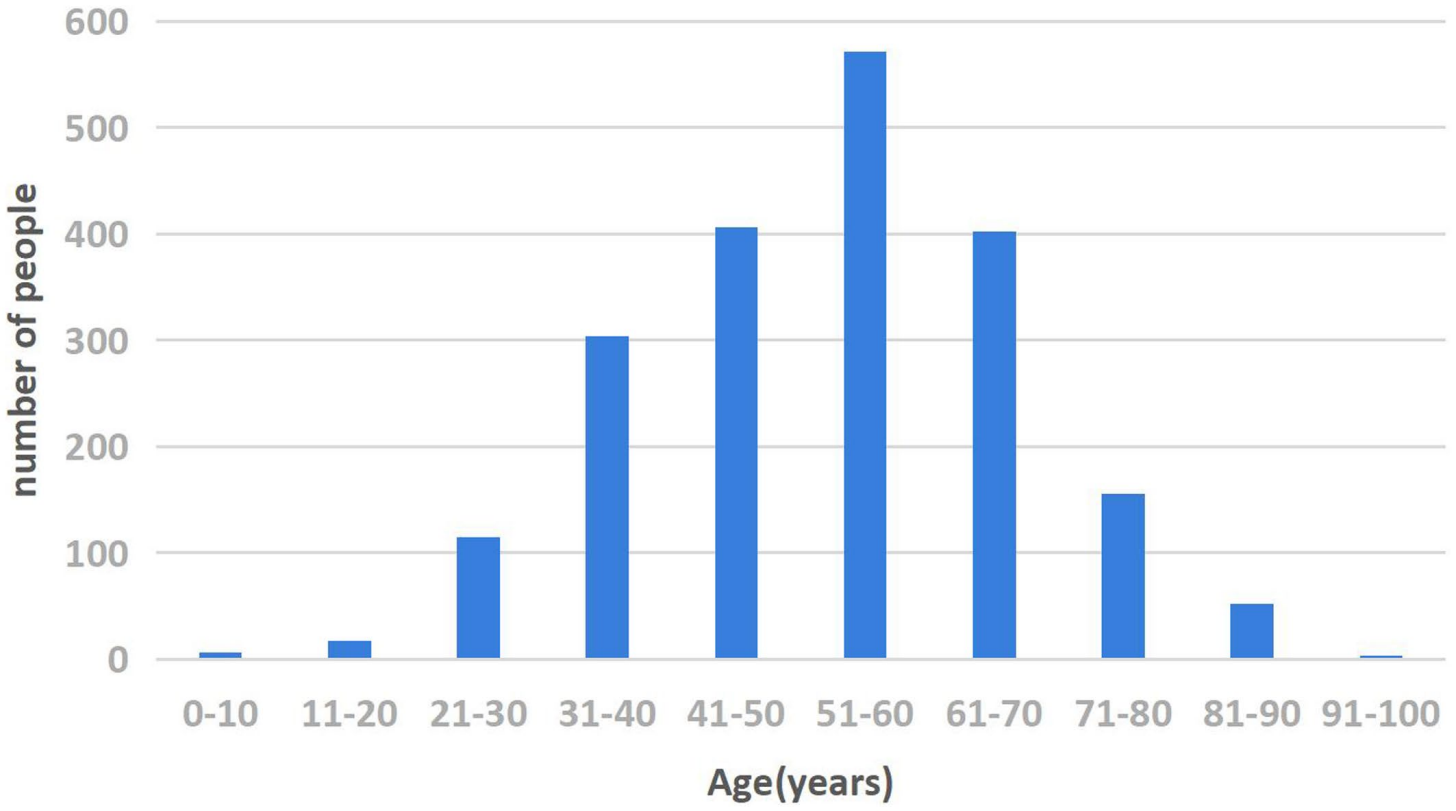
Age distribution of HE in the four hospitals of Hainan Province from 2013 to 2022.

From 2013 to 2022, the total incidence of HE among residents of the four hospitals of Hainan Province was 1,922, with 1,466 cases in males and 456 in females, resulting in a sex ratio of 3.21:1. From 2013 to 2019, the HE incidence increased from 0.134 per 100,000 to 3.207 per 100,000, showing an overall upward trend. The incidence rate rose from 0.13 per 100,000 in 2013 to 3.21 per 100,000 in 2019, decreased slightly in 2020 and 2021, and increased again to 2.25 per 100,000 in 2022 (Table 1). In the province, 1,922 cases of HE were reported, with seven cases in the 0–14-year age group, 1,284 cases in the 15–59-year age group, and 631 cases aged 60 years and above, representing 0.37, 66.80, and 32.83% of the population, respectively. The highest percentage was in the 50–59-year age group (555 cases, 28.88%; Figure 2).

**Table 1:** Collected Hepatitis E cases data and estimated crude incidence rates from 2013 to 2022.

Year	Total population of Hainan Province	The number of collected cases of HE	The crude incidence rate of HE
2013	895.3w	12	1.34/1million
2014	903.5w	65	7.19/1million
2015	910.8w	112	13.00/1million
2016	917.1w	220	23.99/1million
2017	925.8w	258	27.87/1million
2018	934.3w	292	31.25/1million
2019	944.7w	303	32.07/1million
2020	1012.3w	249	24.60/1million
2021	1020.5w	181	17.74/1million
2022	Unavailable	230	Unavailable

The total population data for Hainan Province is obtained from the Hainan Provincial Bureau of Statistics (http://www.hainan.gov.cn/). The incidence rate refers to the ratio of the number of cases to the total population of the province, usually calculated as per 10000. The population data for the year 2022 from the Hainan Provincial Bureau of Statistics is not yet available.

**Figure 3:**
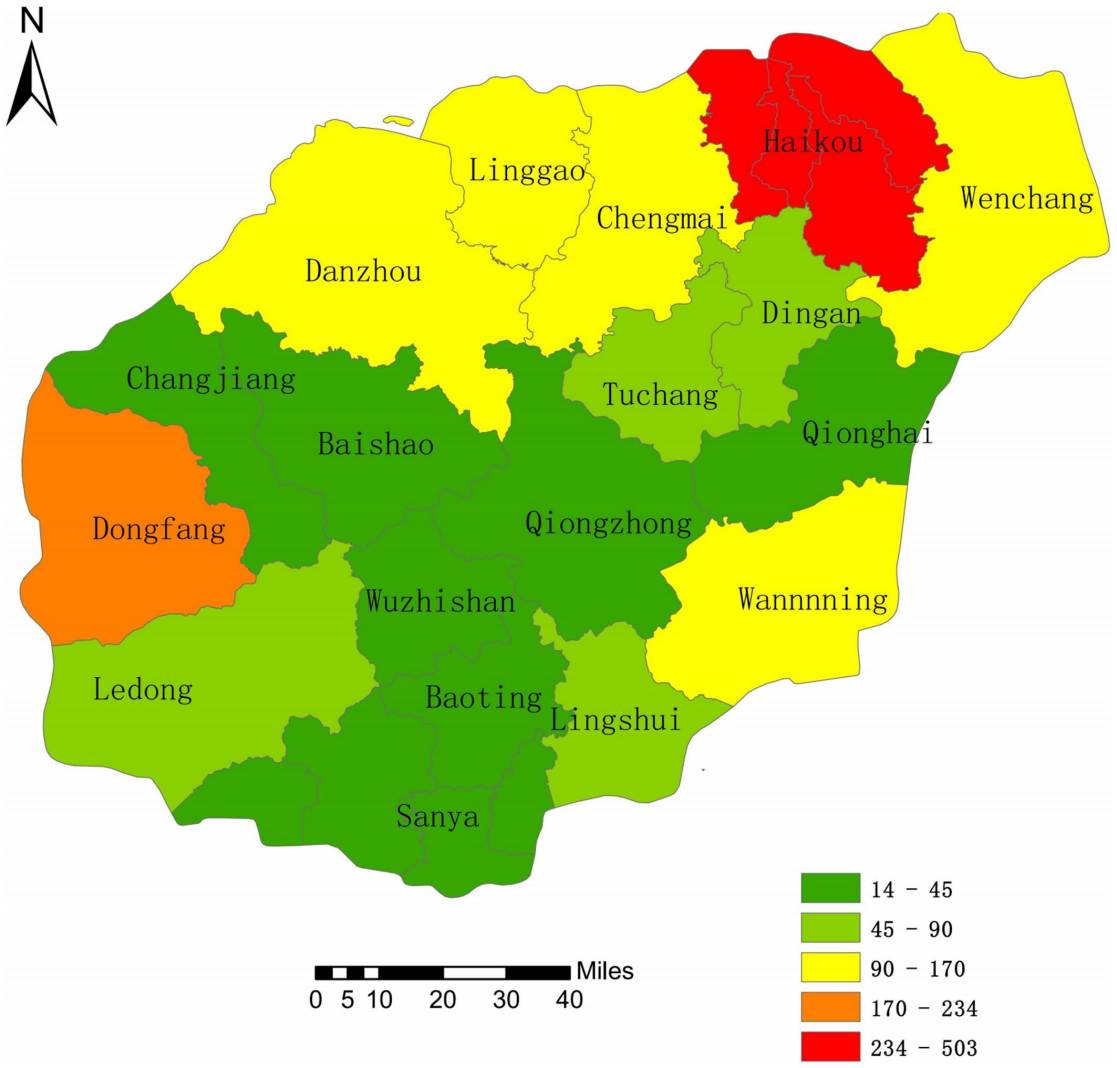
Spatial distribution Map of HE incidence.

From 2013 to 2022, the incidence of HE in the four hospitals of Hainan Province was highest in Haikou, with 498 cases, followed by Dongfang City and Danzhou City, with 234 and 170 cases, respectively. The least affected area was Wuzhishan (14 cases). The five areas with the highest incidence of HE from 2013 to 2022 were Haikou City, Dongfang City, Danzhou City, Wanning City, and Lin Gao County. The incidence was higher in the northwest areas of Hainan Province and lower in the southeast (Figure 3).

**Figure 4:**
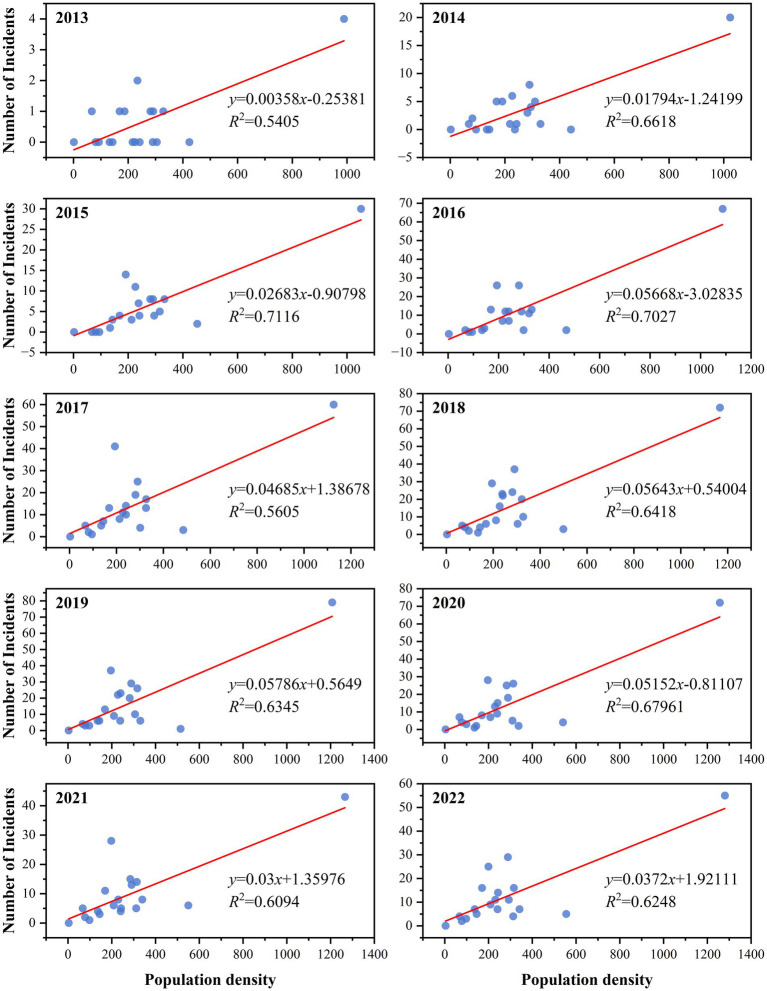
Scatter plot of the correlation between population density and the incidence of hepatitis E from 2013 to 2022.

We conducted a correlation analysis on the influencing factors of morbidity, specifically focusing on population density, economy, and geographical location. We conducted a normality test on the number of reported cases from 2013 to 2022, and the significance *p*-value was less than 0.05, indicating that the data does not follow a normal distribution. The calculation of Spearman correlation coefficients showed that the correlation coefficients (r-values) between morbidity and population density, economy, and geographical location (latitude) are 0.414, 0.421, and 0.403, respectively (Table 2). At the same time, a scatter plot was drawn to show the correlation between population density and incidence from 2013 to 2022, with r^2^ values fluctuating between 0.5405 and 0.7116, indicating a linear correlation (Figure 4).

**Table 2:** Spearman correlation coefficients of population density, economy, geographic location (latitude and longitude), and incidence from 2013 to 2022.

Correlation							
			Incidence	Population density	economy	Longitude	Latitude
Spearman Rho	Incidence	Correlation coefficient	1	0.414^**^	0.421^**^	0.13	0.403^**^
		Significance (two-tailed)		0	0	0.08	0
		Case	183	183	126	183	183
	Population density	Correlation coefficient	0.414^**^	1	0.739^**^	0.370^**^	0.282^**^
		Significance (two-tailed)	0		0	0	0
		case	183	183	126	183	183
	Economy	Correlation coefficient	0.421^**^	0.739^**^	1	0.236^**^	0.089
		Significance (two-tailed)	0	0		0.008	0.324
		Case	126	126	126	126	126
	Longitude	Correlation coefficient	0.13	0.370^**^	0.236^**^	1	0.265^**^
		Significance (two-tailed)	0.08	0	0.008		0
		case	183	183	126	183	183
	Latitude	Correlation coefficient	0.403^**^	0.282^**^	0.089	0.265^**^	1
		Significance (two-tailed)	0	0	0.324	0	
		Case	183	183	126	183	183

**At the 0.01 level (two-tailed), the correlation is significant. The population density and economic data were obtained from the 2023 Hainan Statistical Yearbook. It should be noted that economic data was only updated for the years 2015 and 2017–2022. The longitude and latitude data for each city and county were sourced from Tianditu (https://map.tianditu.gov.cn/). Economic indicators come from local fiscal revenue and expenditure in various cities and counties. Local fiscal revenue includes local general budget revenue and expenditure and fund revenue. Longitude and latitude are the coordinates of the icons in each city and county.

### Global spatial autocorrelation distribution

From 2013 to 2022, Moran’s I index for each year was consistently positive, ranging from 0.40 to 0.57. The year 2018 (Moran’s I = 0.57) had the highest value, whereas 2021 (Moran’s I = 0.40) had the lowest value. This suggests that the data were not randomly distributed and exhibited a significant positive spatial autocorrelation, indicating a clear clustered distribution (Table 3).

**Table 3:** Moran’s I values for the overall spatial autocorrelation of HE incidence rate in Hainan Province from 2013 to 2022.

Year	Moran’s I	*z*-value	*p*-value	Cluster
2013	0.419429	3.633609	0.000279	Yes
2014	0.509051	4.365732	0.000013	Yes
2015	0.566876	4.811193	0.000002	Yes
2016	0.496218	4.271927	0.000019	Yes
2017	0.455212	3.887422	0.000101	Yes
2018	0.567898	4.787997	0.000002	Yes
2019	0.517095	4.406162	0.000011	Yes
2020	0.517064	4.43019	0.000009	Yes
2021	0.40486	3.510893	0.000447	Yes
2022	0.458299	3.943711	0.00008	Yes

The Moran’s I value ranges between (−1, 1), where Moran’s I > 0 indicates positive spatial autocorrelation, with larger values implying stronger spatial correlation. Moran’s I < 0 indicates negative spatial autocorrelation, with smaller values suggesting greater spatial dissimilarity. When Moran’s I = 0, spatial distribution is considered random. The *Z*-value represents the number of standard deviations, with Z > 1.65 indicating a clustered distribution, *Z* < 1.65 indicating a dispersed distribution, and values in between suggesting a random distribution. The P-value represents the significance level of the hypothesis test. When *P* < 0.01, it indicates rejection of the null hypothesis at a 99% confidence level, implying the presence of spatial autocorrelation.

### Local spatial autocorrelation distribution

**Figure 5:**
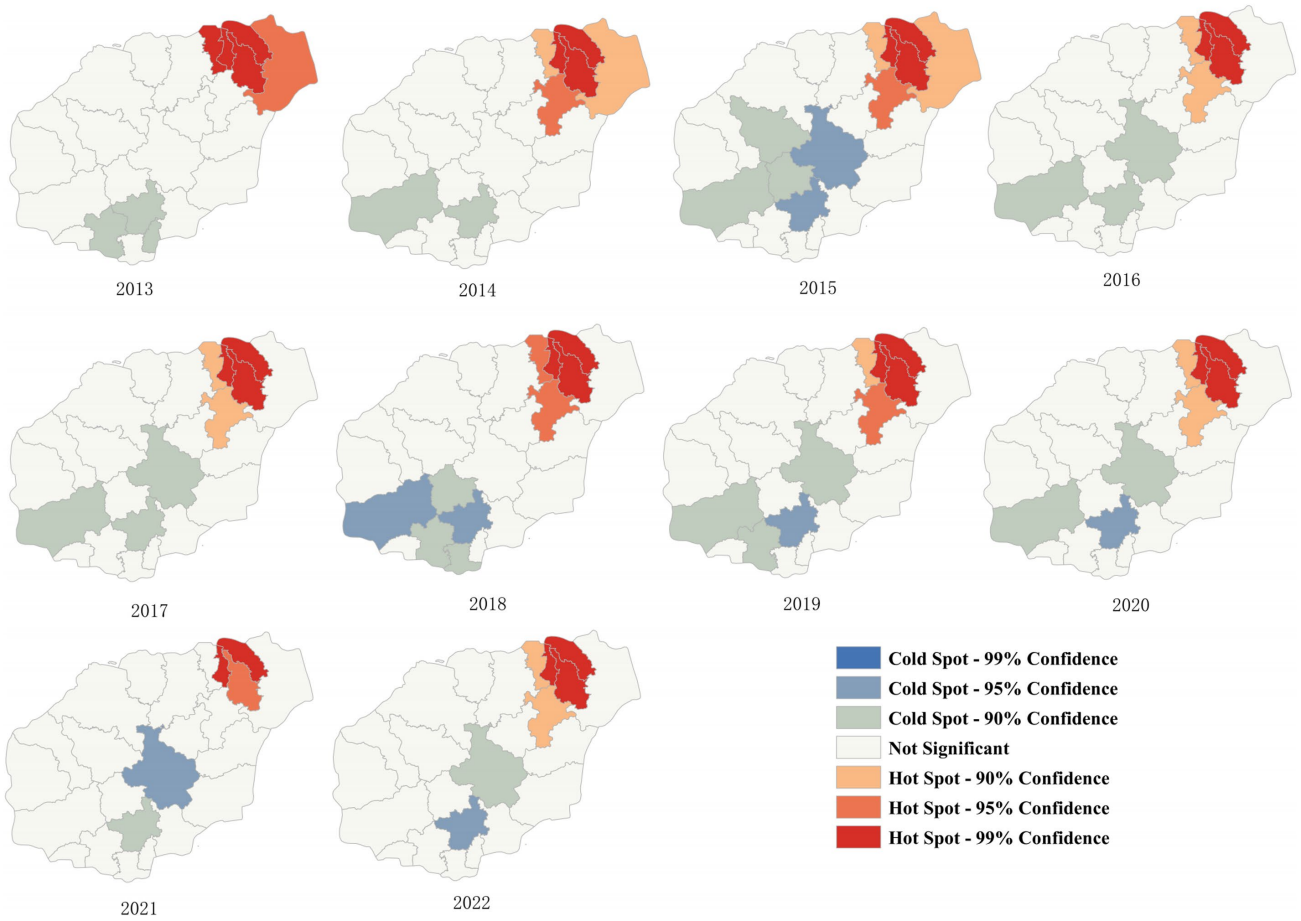
LISA of the four hospitals of Hainan Province from 2013 to 2022.

From 2013 to 2022, the average density of HE incidence in the four hospitals of Hainan Province revealed seven clusters. During this period, the incidence was concentrated in the northern region (Figure 5). HE hotspots varied yearly from 2013 to 2022. In 2013, the hotspots were Haikou and Wenchang, with Baoting being the only cold-spot. In 2014, the hotspots were Haikou, Wenchang, Chengmai, and Ding’an, with cold spots in Ledong and Baoting. In 2015, the hotspots were the same as those in 2014, with cold spots in Wuzhishan, Baisha, Ledong, Baoting, and Qiongzhong. In 2016 and 2017, the hotspots were Haikou and Ding’an, with cold spots in Ledong, Baoting, and Qiongzhong. In 2018, the hotspots were Haikou and Ding’an, with cold spots in Ledong, Baoting, and Sanya. In 2019 and 2020, the hotspots were Haikou and Ding’an, with cold spots in Ledong, Baoting, and Sanya. In 2021, the hotspot was Haikou, with cold spots in Baoting and Qiongzhong. In 2022, the hotspots were Haikou and Ding’an, with cold spots in Baoting and Qiongzhong (Figure 5).

### Central tendency and dispersion analysis

**Figure 6:**
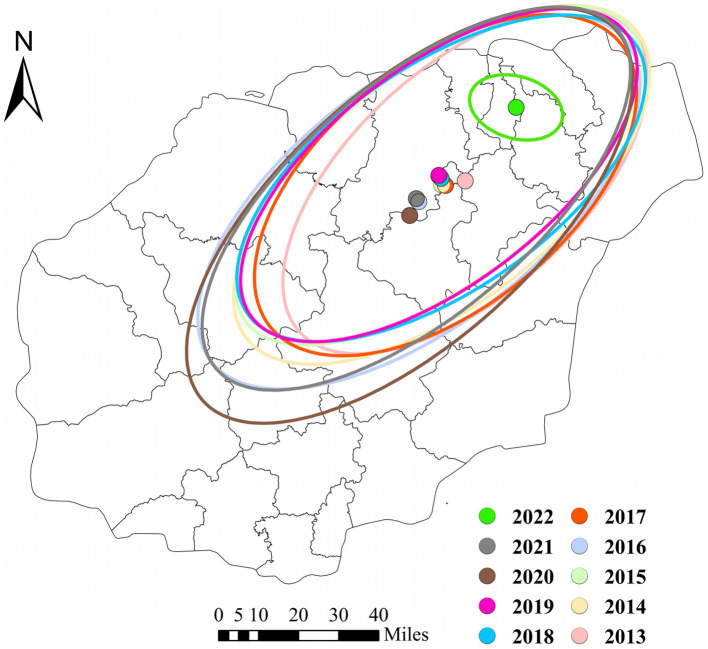
Changes in the Center and Dispersion Trend of Average Density of HE Cases in the four hospitals of Hainan Province from 2013 to 2022.

Figure 6 shows the changes in the average center and standard deviation ellipse of the average density distribution of HE incidence cases in Hainan Province from 2013 to 2022. The average center of the density distribution of HE cases in Hainan Province initially moved from the northeast to the southwest in 2013, continued to move from the northeast to the southwest after 2015, shifted from the northeast to the southwest again after 2019, and moved back to the northeast by 2022. From 2013 to 2021, the distribution was mainly concentrated at the junction of Chengmai, Tunchang, and Ding’an Counties, whereas in 2022, it was mainly concentrated in Haikou City.

### Time-series model forecasting

**Figure 7:**
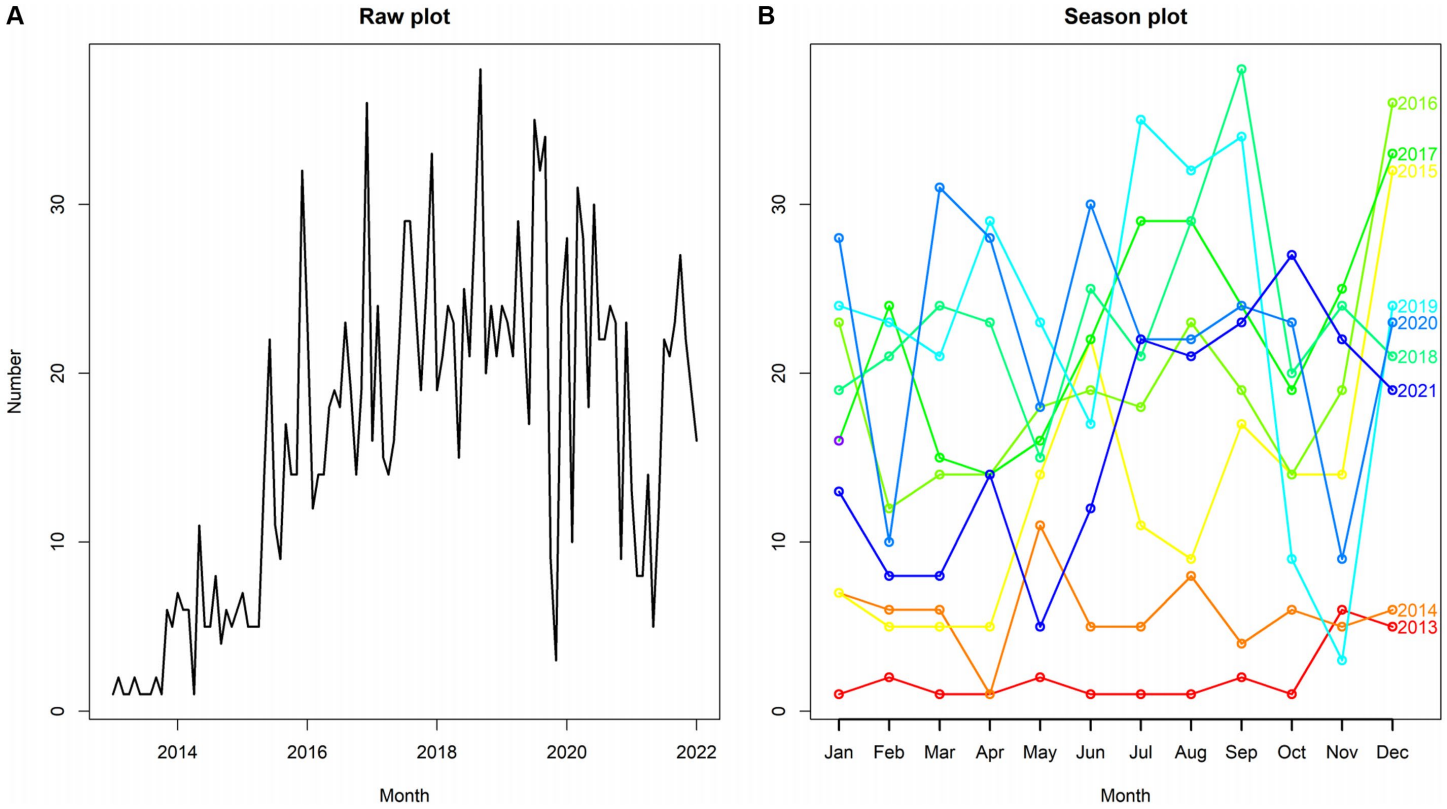
Time series chart of hepatitis E incidence in the four hospitals of Hainan Province from 2013 to 2022.

**Figure 8:**
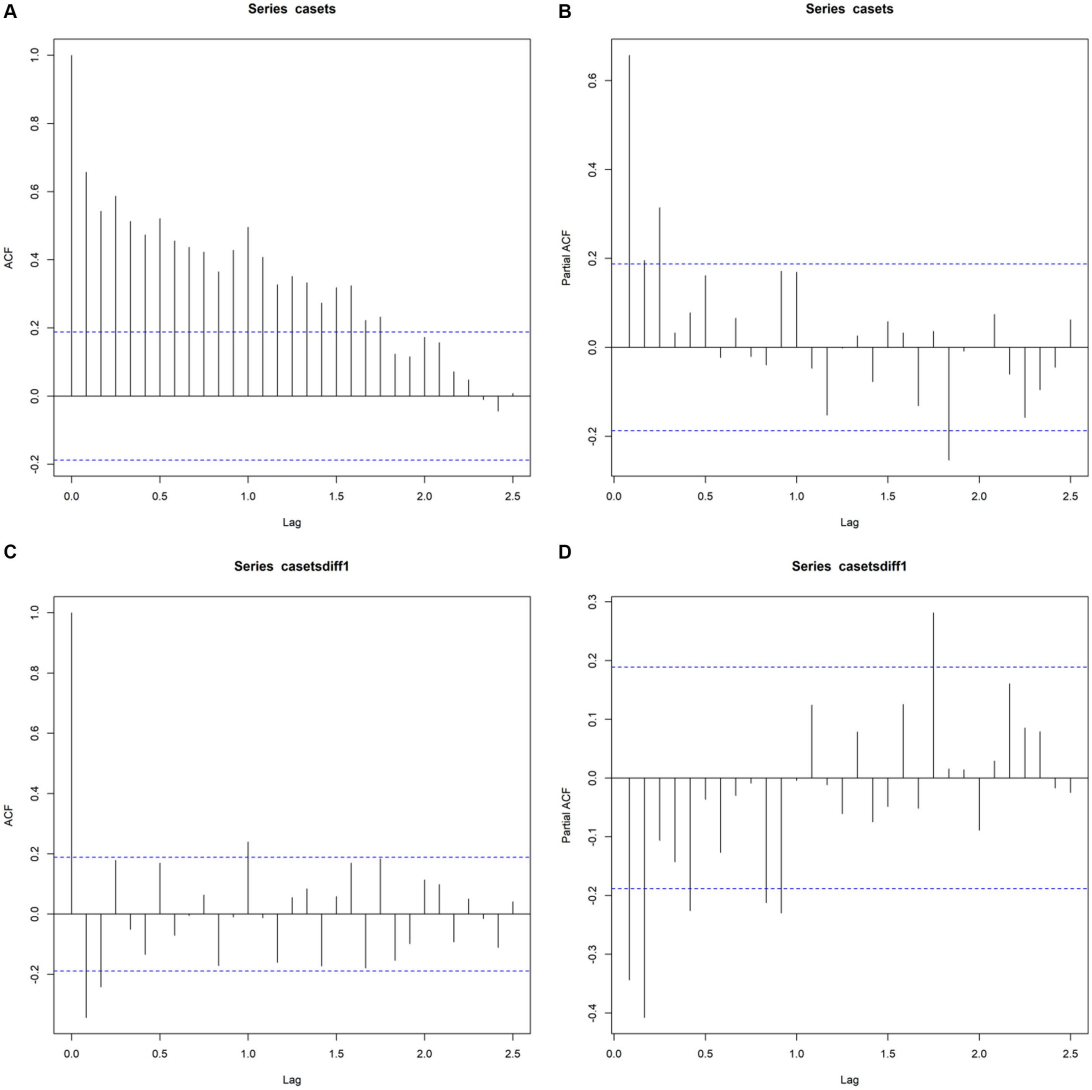
Autocorrelation and partial autocorrelation plots before and after differencing **(A,B)**, represents ACF and PACF plots of the original sequence, and **(C,D)** represents ACF and PACF plots after first-order differencing.

**Figure 9:**
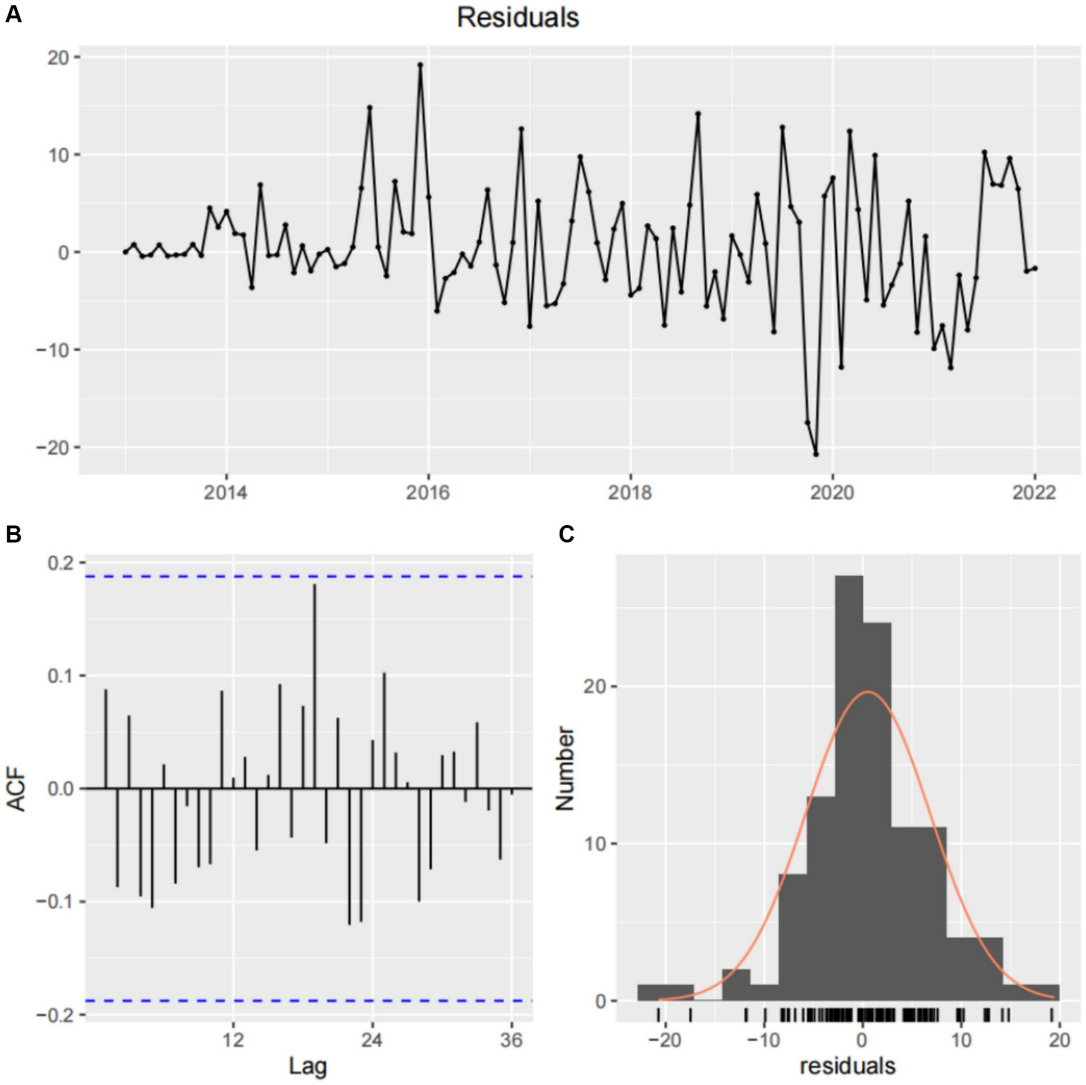
White noise test after differencing and normal distribution of model residuals. **(A)** Residual sequence plot. **(B)** Model residual autocorrelation diagram. **(C)** Normal distribution plot of model residuals.

**Figure 10:**
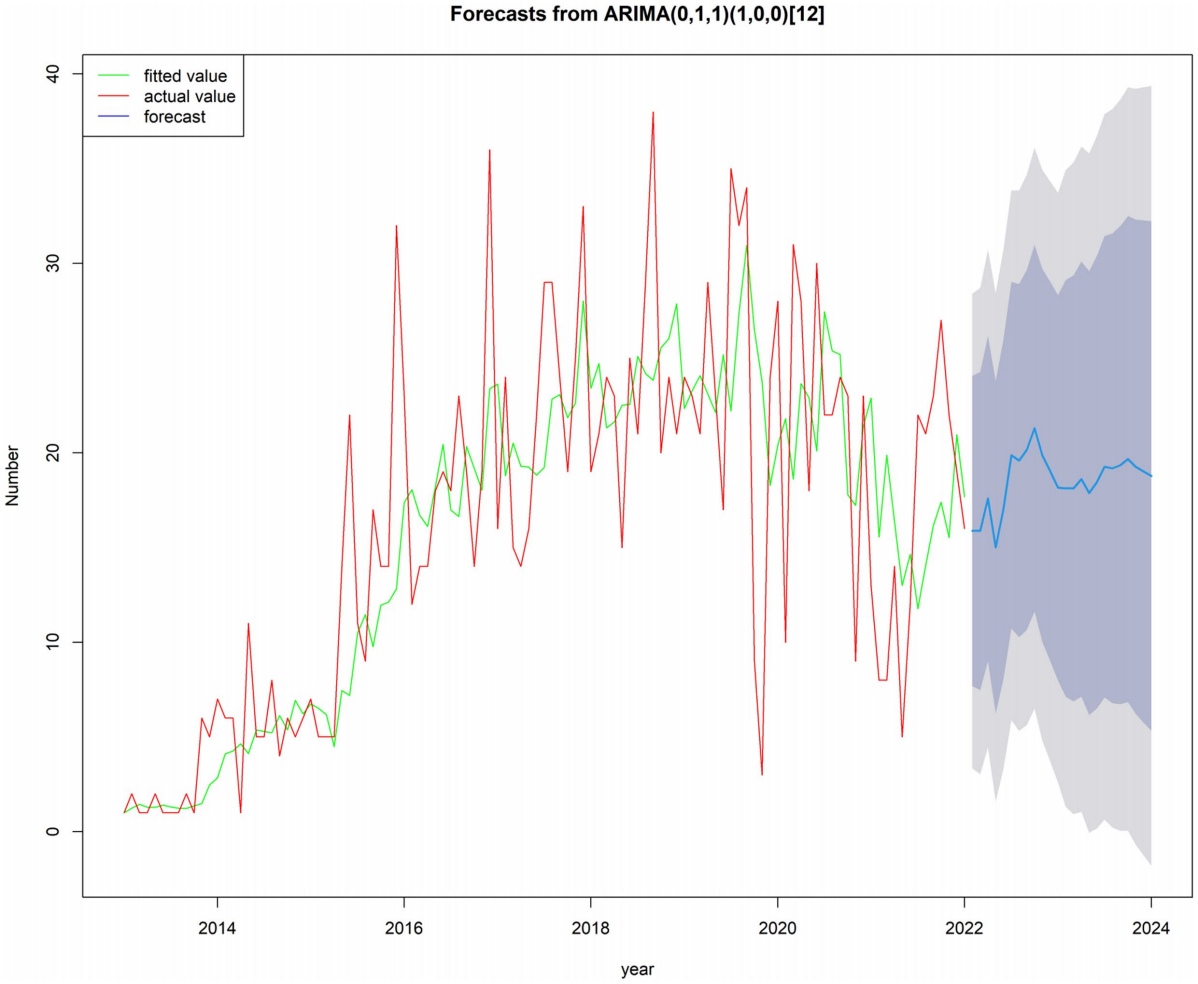
Time series plot: future 24 months based on incidence data from January 2013 to December 2022.

The reported incidence of HE in the four hospitals of Hainan Province increased from 12 cases in 2013 to 230 cases in 2022, with cases reported throughout all four seasons, constituting a seasonal cycle of 12 months. As shown in the time-series plot of HE incidence in the four hospitals of Hainan Province from 2013 to 2022 (Figure 7), there is no clear seasonal peak or regularity in the number of HE cases, indicating a non-stationary sequence (P for stationary test before differencing = 0.3922). After adjustment, the autocorrelation and partial autocorrelation coefficients were within the 95% confidence interval (CI). The autocorrelation function exhibits a rapid decay, suggesting that the adjusted series is similar to a stationary sequence (P for the stationary test after first-order differencing = 0.01). Figure 8 shows the autocorrelation and partial autocorrelation plots before and after differencing, confirming that the time series meets the requirements of a stationary non-white noise sequence. Using the “auto. Arima” function in R, based on AIC for automatic parameter selection, the model with ARIMA (0,1,1)(1,0,0)[12] had the lowest AIC value of 713.2863, indicating that the preliminary model is an ARIMA (0,1,1)(1,0,0)[12] model with a 12-month cycle. The parameters of the ARIMA (0,1,1)(1,0,0)[12] model are specified as follows: *p* = 0, d = 1, and q = 1, indicating a differencing order of 1 and a moving average order of 1. (1,0,0) denotes a seasonal differencing order of 1, indicating a seasonal difference at the 12-time points (Table 4). The Ljung-Box test was used to evaluate the model’s fitting effect (*p* > 0.05), with Q = 17.378 and *p* = 0.7422, indicating that the established model parameters were statistically significant, with the white noise test passed (Figure 9). Therefore, the fitted model is considered effective, and the predicted values suggest that the monthly newly reported cases will be relatively stable in 2023 and 2024, fluctuating between 17 and 19 cases (Table 5). Visualization of the prediction results showed that the 95% CI of the true values was within the predicted range, indicating that the dynamic trend of the prediction model was generally consistent with the actual incidence (Figure 10).

**Table 4:** Model selection process table.

ARIMA model	AIC values
ARIMA(2,1,2) (1,0,1)[12]	720.2358
ARIMA(0,1,0)	756.5758
ARIMA(1,1,0) (1,0,0)[12]	737.7884
ARIMA(0,1,1) (0,0,1)[12]	715.6168
ARIMA(0,1,0)	754.5328
ARIMA(0,1,1)	720.8844
ARIMA(0,1,1) (1,0,1)[12]	716.557
ARIMA(0,1,1) (0,0,2)[12]	716.5319
ARIMA(0,1,1) (1,0,0)[12]	714.5192
ARIMA(0,1,1) (2,0,0)[12]	716.5521
ARIMA(0,1,1) (2,0,1)[12]	718.7507
ARIMA(0,1,0) (1,0,0)[12]	752.6328
ARIMA(1,1,1) (1,0,0)[12]	714.682
ARIMA(0,1,2) (1,0,0)[12]	714.342
ARIMA(0,1,2)	719.6684
ARIMA(0,1,2) (2,0,0)[12]	716.374
ARIMA(0,1,2) (1,0,1)[12]	716.3986
ARIMA(0,1,2) (0,0,1)[12]	715.3597
ARIMA(0,1,2) (2,0,1)[12]	718.5127
ARIMA(1,1,2)(1,0,0)[12]	715.6917
ARIMA(0,1,3) (1,0,0)[12]	716.0693
ARIMA(1,1,3) (1,0,0)[12]	717.9733
ARIMA(0,1,2) (1,0,0)[12]	713.3163
ARIMA(0,1,2)	719.1172
ARIMA(0,1,2) (2,0,0)[12]	715.1982
ARIMA(0,1,2) (1,0,1)[12]	715.2127
ARIMA(0,1,2) (0,0,1)[12]	714.5137
ARIMA(0,1,2) (2,0,1)[12]	717.3122
ARIMA(0,1,1) (1,0,0)[12]	713.2863
ARIMA(0,1,1)	720.1083
ARIMA(0,1,1) (2,0,0)[12]	715.1987
ARIMA(0,1,1) (1,0,1)[12]	715.1932
ARIMA(0,1,1) (0,0,1)[12]	714.5535
ARIMA(0,1,1) (2,0,1)[12]	717.3675
ARIMA(0,1,0) (1,0,0)[12]	750.544
ARIMA(1,1,1) (1,0,0)[12]	713.6361
ARIMA(1,1,0) (1,0,0)[12]	735.6859
ARIMA(1,1,2) (1,0,0)[12]	714.5941
Best model: ARIMA(0,1,1)(1,0,0)[12]	

**Table 5:** Projected monthly incidence of hepatitis E in Hainan Province, 2023–2024.

Point		Forecast	Lower CI 80%	Higher CI 80%	Lower CI 95%	Higher CI 95%
Jan	2023	18.16	7.983	28.336	2.596	33.724
Feb	2023	18.124	7.129	29.118	1.309	34.939
Mar	2023	18.124	6.883	29.365	0.932	35.316
Apr	2023	18.614	7.131	30.096	1.053	36.174
May	2023	17.879	6.16	29.598	−0.043	35.801
Jun	2023	18.45	6.5	30.401	0.174	36.727
Jul	2023	19.267	7.089	31.444	0.643	37.89
Aug	2023	19.185	6.785	31.585	0.22	38.15
Sep	2023	19.348	6.729	31.968	0.048	38.648
Oct	2023	19.675	6.84	32.51	0.045	39.304
Nov	2023	19.267	6.22	32.313	−0.687	39.22
Dec	2023	19.022	5.767	32.277	−1.25	39.294
Jan	2024	18.777	5.317	32.237	−1.809	39.363
Feb	2024	18.767	4.974	32.559	−2.328	39.861
Mar	2024	18.767	4.751	32.782	−2.669	40.202
Apr	2024	18.907	4.671	33.142	−2.864	40.677
May	2024	18.697	4.245	33.148	−3.405	40.798
Jun	2024	18.86	4.196	33.524	−3.567	41.287
Jul	2024	19.093	4.219	33.967	−3.655	41.841
Aug	2024	19.07	3.989	34.151	−3.995	42.134
Sep	2024	19.116	3.831	34.402	−4.26	42.493
Oct	2024	19.21	3.723	34.696	−4.475	42.895
Nov	2024	19.093	3.407	34.779	−4.896	43.082
Dec	2024	19.023	3.141	34.905	−5.266	43.313

## Discussion

We analyzed HE incidence in the four hospitals of Hainan Province from 2013 to 2022 and performed a descriptive analysis considering time, region, age, and sex to elucidate the distribution and epidemiological characteristics of HE in the region. The research findings indicate that males are more susceptible to HE than females, which is consistent with previous research results (25), and may be associated with higher occupational exposure and greater participation in social activities among males (26). The incidence occurs across different age groups, primarily affecting middle-aged and older adults. Studies have suggested a close relationship between age and the presence of anti-HEV IgG antibodies, reflecting potential cumulative exposure to the virus (9). Multiple studies have also confirmed the possibility of long-term preservation of anti-HEV antibodies after exposure (8, 9, 27). However, this is insufficient to explain why the incidence of HE is higher in middle-aged and older adults. Therefore, further epidemiological and laboratory studies are required to confirm the reasons for the higher incidence in this age group. The overall trend of HE incidence in Hainan Province showed an initial increase followed by a decrease, consistent with the trend observed in previous studies in other regions of the country (15). The increase in HE incidence could be attributed to implementing measures to control the COVID-19 pandemic, resulting in reduced social activities and decreased cases compared with previous periods. The reasons for the elevated HE incidence are as follows: (1) With rapid economic growth and the development of the tourism and catering industry, there has been an increase in population mobility, leading to a higher chance of human-source infections (28); (2) With the improvement of medical conditions, there has been an increase in the rate of testing and higher diagnostic levels, and research indicates that the increase in the rate of diagnosed HE cases closely corresponds to the increase in the rate of diagnostic test kits sold in the Chinese market (29); and (3) With the advent of electronic medical record systems, there has been an enhancement in doctor reporting awareness (30, 31), with various medical institutions strengthening the quality of infectious disease reporting and reducing the number of underreported cases (32, 33).

From the thematic maps of HE incidence in this study, it is evident that the northern regions of Hainan Province, particularly Haikou City, are focal points for disease occurrence. This may be due to the large population, high density, significant population movement, and multiple ports in these urban areas (34, 35). The higher incidence in these areas may be related to increased economic and cultural exchange activities. Research suggests that medical resources, economic factors, ethnicity, and provincial background can influence disease spread (36). The occurrence and distribution of diseases are closely associated with geographical and meteorological factors (22, 37). Spatial epidemiology is widely applied in the monitoring of infectious diseases, particularly in analyzing spatial distribution patterns and regional clustering of infectious diseases (38). Analyzing the spatial autocorrelation of infectious diseases makes it possible to quickly identify regions with disease occurrence and recognize spatial clustering. This provides a scientific basis for developing measures to prevent and control infectious diseases (38, 39). The global spatial autocorrelation results of this study indicated that Moran’s I values for HE incidence in the province from 2013 to 2022 were consistently positive, suggesting that disease occurrence during this period was not randomly distributed. A significant positive spatial autocorrelation indicated a clear clustered distribution of HE cases (*p* < 0.01, statistically significant difference). The local spatial autocorrelation results showed that the hotspots of HE incidence from 2013 to 2022 were mostly concentrated in the economically developed northern region. Through concentration trends and dispersion analysis, the results suggested a clear directionality in HE incidence, indicating a trend of spreading toward the southern part of the province. The northern region, with its high population density and strong population mobility, coupled with the development of the catering and tourism industries, may be related to the increased incidence and transmission rate of HE (19).

Time-series forecasting analysis can incorporate various influencing factors, including the combined effects of unknown components, into the time variable (22). The ARIMA model is used to prevent and control various legally reportable infectious diseases (40–42). The forecast results of the ARIMA(0,1,1)(1,0,0)[12] model in this study indicate that newly reported cases are relatively stable, fluctuating between 17 and 19 cases per month in 2023 and 2024. Although the risk of HE transmission persists, the likelihood of short-term outbreaks appears to be low.

Our study had several limitations. First, the data we selected came from four hospitals in Haikou City that detected HE over the past decade. We only used the HE cases reported in the hospital system for statistical analysis and did not obtain data from some potential carriers or asymptomatic patients, which may lead to selection bias. However, these patients usually do not seek medical attention or testing, so even the data from the disease control department cannot detect this portion of the infected. In the future, we can adopt sampling survey methods to test HE antibodies in people from various regions to understand their prevalence levels. Second, the incidence of HE is influenced not only by time but also by factors such as population, social activities, economy, climate, and the environment. This study did not collect and analyze potential factors that may affect the distribution of HE incidence. The data were updated as time progressed, and the established model was not static. Therefore, we need to continue to update the HE incidence data, constantly revise the model parameters, and improve the prediction accuracy. At the same time, incorporating factors that affect the incidence of HE into the prediction model will help us obtain a more accurate HE prediction model. Thirdly, our research did not investigate the relevant factors that affect clustering.

## Conclusion

From 2013 to 2019, the incidence of HE in Hainan Province increased. The incidence rate was significantly higher in males than in females, and the distribution of incidence rates across all age groups tended to be higher in middle-aged adults, with lower rates in younger and older age groups, and the incidence was highest in the 50–59-year age group. The areas with the highest incidence were mainly concentrated in the northeast, with a tendency to gradually spread toward the southwest over time. The HE status in Hainan Province from 2013 to 2024 remained relatively stable, and the ARIMA model accurately predicted the epidemic. This model can be used for short-term forecasting of HE incidence and predicts a relatively stable number of new cases each month in 2023 and 2024.

## Data availability statement

The original contributions presented in the study are included in the article/Supplementary material, further inquiries can be directed to the corresponding author.

## Ethics statement

This study was approved by the Research Ethics Committee of Hainan General Hospital (No. 2021-241). Written informed consent was not required as the data was anonymized for the study. Consent was waived by the Research Ethics Committee.

## Author contributions

ZY: Writing – original draft, Methodology, Data curation, Conceptualization. P-PL: Writing – review & editing, Formal analysis. J-ZW: Writing – review & editing, Formal analysis. FL: Writing – review & editing, Methodology, Data curation, Conceptualization. W-TL: Writing – review & editing, Formal analysis. M-HW: Writing – review & editing, Formal analysis. Y-RZ: Writing – review & editing, Formal analysis. H-ZW: Writing – review & editing, Formal analysis. HL: Writing – review & editing, Formal analysis. X-FC: Writing – review & editing, Formal analysis. X-BL: Writing – review & editing, Formal analysis. X-XF: Writing – review & editing, Formal analysis. TW: Writing – review & editing, Project administration, Methodology, Data curation. YG: Writing – review & editing, Project administration, Methodology, Data curation, Conceptualization.

## Footnotes

^1^ http://www.nhc.gov.cn/jkj/

^2^ https://www.chinacdc.cn/images/jlogo.png

^3^ http://www.hainan.gov.cn/

^4^ https://hainan.tianditu.gov.cn/

^5^ https://map.tianditu.gov.cn/
